# The Synergistic Risk of Insulin Resistance and Renal Dysfunction in Acute Coronary Syndrome Patients After Percutaneous Coronary Intervention

**DOI:** 10.3390/jcdd12110427

**Published:** 2025-10-28

**Authors:** Guoshu Yang, Maoling Jiang, Lin Liu, Dongyue Jia, Jie Feng, Yan Luo, Tao Ye, Long Xia, Hanxiong Liu, Zhen Zhang, Jinjuan Fu, Lin Cai, Qiang Chen, Shiqiang Xiong

**Affiliations:** 1 Department of Cardiology, The Third People’s Hospital of Chengdu, Chengdu 610014, China; guoshuy@126.com (G.Y.); mmmaoling@163.com (M.J.); jiadongyue2023@163.com (D.J.); fj00127@163.com (J.F.); luoyan31566@163.com (Y.L.); 15298272185@163.com (T.Y.); 15928489306@163.com (L.X.); lhanx@126.com (H.L.); zhangzhen@swjtu.edu.cn (Z.Z.); fujinjuan@swjtu.edu.cn (J.F.); clin63@yeah.net (L.C.); 2 Department of Integrated Traditional Chinese and Western Internal Medicine, The General Hospital of Central Theater Command, Wuhan 430000, China; doctorliu2024@126.com; 3 Institute of Clinical Medical Sciences, China-Japan Friendship Hospital, Chinese Academy of Medical Sciences, Peking Union Medical College, Beijing 100029, China

**Keywords:** insulin resistance, renal function, acute coronary syndrome, percutaneous coronary intervention, prognosis

## Abstract

**Background:** Despite percutaneous coronary intervention (PCI) for revascularization, patients with acute coronary syndrome (ACS) still face residual risks of adverse outcomes. Insulin resistance (IR) and renal impairment are independent predictors of poor prognosis in these patients, yet their interaction and underlying mechanisms linked to post-PCI outcomes remain incompletely elucidated. **Methods:** A retrospective cohort study was conducted involving patients with ACS who underwent PCI at the Third People’s Hospital of Chengdu from July 2018 to December 2020. Insulin resistance (IR) was quantified using the triglyceride–glucose (TyG) index, and renal function was evaluated via the estimated glomerular filtration rate (eGFR). The primary endpoint was major adverse cardiovascular events (MACEs), a composite of all-cause death, non-fatal myocardial infarction, non-fatal stroke, and unplanned revascularization. Multivariable Cox proportional hazards regression and mediation analyses were applied to explore the associations of TyG index and eGFR with patient prognosis, and to quantify the mediating effect of eGFR on the relationship between TyG index and prognosis. **Results:** A total of 1340 patients with ACS were included in the final analysis. Over a median follow-up duration of 31.02 (interquartile range [IQR]: 27.34–35.03) months, 124 patients (9.25%) experienced MACEs. After adjusting for potential confounders, both the TyG index and eGFR were identified as significant independent predictors of MACEs in the overall population and across predefined subgroups. Specifically, each one-unit increase in the TyG index was associated with a 73.8% higher risk of MACEs (HR 1.738; 95% CI 1.273–2.372), whereas each ten-unit decrease in eGFR was linked to a 12.7% increased MACEs risk (HR 1.127; 95% CI 1.032–1.232). Importantly, after further adjustment for confounders, eGFR significantly mediated 9.63% of the total effect of the TyG index on MACEs risk. **Conclusions:** Renal impairment partially mediates the association between IR and adverse cardiovascular outcomes in ACS patients undergoing PCI. This finding underscores the clinical importance of the metabolic–cardiorenal axis in this population, suggesting that a comprehensive assessment targeting both IR and renal function-related pathways may enhance risk-stratification accuracy and optimize therapeutic strategies for ACS patients.

## 1. Background

Patients with acute coronary syndrome (ACS) still face high long-term risk of major adverse cardiovascular events (MACEs) despite percutaneous coronary intervention (PCI) [1,2]. Thus, early risk stratification is clinically valuable for enhancing their long-term prognosis. Insulin resistance (IR) is a key driver of type 2 diabetes, atherosclerosis, and metabolic disorders and is strongly linked to cardiovascular disease progression and adverse outcomes [3,4,5]. The triglyceride–glucose index (TyG index), which evaluates the body’s insulin sensitivity by fasting blood glucose and triglyceride, has been validated as a simple and reliable alternative indicator of IR [6,7] and is now widely used in clinical research [8]. Recent studies have shown that an elevated TyG index is independently associated with the incidence and progression of various cardiovascular diseases, including myocardial infarction [9], coronary artery calcification [10,11], peripheral artery disease [12], and stroke [13].

Renal impairment is also recognized as a well-established risk factor for cardiovascular disease, including coronary artery disease (CAD), heart failure (HF), arrhythmias, and sudden cardiac death [14,15]. Renal impairment exacerbates cardiovascular risk through mechanisms such as uremic toxin accumulation, chronic inflammation, and fluid overload [16]. Recent research has confirmed that the risk for cardiovascular events increases with declining estimated glomerular filtration rate (eGFR) [17], and reduced eGFR was independently associated with medium-term mortality in ACS patients [18].

Critically, IR and renal impairment are pathophysiologically bidirectionally linked: IR promotes renal damage via glomerular hyperfiltration and fibrosis, while renal impairment exacerbates IR through altered insulin metabolism [19,20]. A national cohort study from China demonstrated that renal impairment mediates the association between IR and the incidence of cardiovascular disease in a general healthy population [21]. However, the study focused on a healthy Chinese population aged over 45 years, and its findings may not be fully generalizable to broader or diseased populations. Thus, it remains unclear whether the “insulin resistance–renal impairment-cardiovascular events” pathway is still significant in ACS patients following PCI.

Therefore, this study aimed to investigate the relationship between IR (assessed by the TyG index) and renal impairment (evaluated by eGFR) with long-term adverse outcomes in ACS patients following PCI, and to verify the potential mediating role of renal impairment in this association.

## 2. Methods

### 2.1. Study Population

The study consecutively enrolled patients hospitalized at the Third People’s Hospital of Chengdu (Chengdu, Sichuan, China) from July 2018 to December 2020. The inclusion criteria were as follows: (1) age ≥ 18 years; and (2) confirmed diagnosis of ACS according to standard clinical criteria and undergoing PCI. The exclusion criteria included the following: (1) loss to follow-up; (2) presence of severe underlying diseases; (3) diagnosis of stable angina pectoris; (4) incomplete critical medical data exceeding 10%; and (5) in-hospital death. A total of 1340 patients were included in the final analysis. This study was approved by the institutional review board and adhered to the Declaration of Helsinki. Informed consent was obtained from all participants.

### 2.2. Data Collection and Definitions

Sociodemographic and clinical data were extracted from electronic medical records. Variables collected included age, sex, height, weight, smoking status, medical history, systolic blood pressure (SBP), heart rate, laboratory results, left-ventricular ejection fraction (LVEF), diagnostic details, and medication use. ACS encompassed conditions such as unstable angina (UA), ST-segment elevation myocardial infarction (STEMI), and non-ST-segment elevation myocardial infarction (NSTEMI), diagnosed according to contemporary guidelines [22]. Medical history included prior PCI, chronic obstructive pulmonary disease (COPD), hypertension, diabetes mellitus, and stroke. Hypertension was defined as SBP ≥ 140 mmHg, diastolic blood pressure (DBP) ≥ 90 mmHg, or current use of anti-hypertensive medication [23]. Diabetes mellitus was diagnosed based on any of the following criteria: fasting glucose ≥ 7 mmol/L, random venous glucose ≥ 11.1 mmol/L, glycated hemoglobin (HbA1c) ≥ 6.5%, 2 h oral glucose tolerance test (OGTT) ≥ 11.1 mmol/L, or current use of anti-diabetic medication [24]. Body mass index (BMI) was calculated as body weight (kg)/height^2^ (m^2^).

### 2.3. Assessment of TyG Index and SYNTAX Score

The TyG index was calculated using the following formula: ln [TG (mg/dL) × FBG (mg/dL)/2]. The baseline SYNTAX score (bSS) was determined using an online calculator (http://syntaxscore.com/, accessed on 1 April 2023), with two independent evaluators analyzing the preprocedural angiograms without knowledge of initial clinical features and outcomes. Discrepancies were resolved by a third evaluator. All data were entered into a dedicated database and assessed for quality.

### 2.4. Outcome Definition

All patients were followed up at 1, 6, and 12 months after discharge, and annually thereafter. The primary endpoint was major adverse cardiovascular events (MACEs), a composite of all-cause death, non-fatal myocardial infarction, non-fatal stroke, and unplanned revascularization. The secondary endpoint included all-cause death, cardiac death, non-fatal myocardial infarction, non-fatal stroke, and unplanned revascularization. All endpoints were adjudicated by experienced clinicians and verified against medical records when available.

### 2.5. Statistical Analyses

Continuous variables are presented as mean ± standard deviation (SD) or median with interquartile range (IQR), based on distribution normality. Group comparisons were made using Student’s t-test or the Mann–Whitney U test, as appropriate. Categorical variables are summarized as frequencies and percentages, and comparisons between groups were performed using the chi-square test or Fisher’s exact test. Correlations among variables were evaluated using Pearson’s correlation coefficients and visualized via a heatmap. The cumulative incidence of adverse cardiovascular outcomes in different groups was estimated using the Kaplan–Meier method, stratified by TyG index and eGFR groups, with between-group differences assessed by log-rank tests.

The dose–response relationships of eGFR and the TyG index with MACEs risk were illustrated using restricted cubic splines (RCS) curves. Cox proportional hazards models were employed to assess associations between the TyG index, eGFR, and adverse cardiovascular outcomes. Hazard ratios (HRs) with 95% confidence interval (CI) were calculated within a time-to-event framework. Subgroup analyses were conducted to evaluate the consistency of the predictive utility of the TyG index and eGFR across different demographic and clinical characteristics. Mediation analysis was performed using VanderWeele’s two-stage regression method for survival data [5] to assess the extent to which eGFR mediates the association between the TyG index (exposure) and MACEs (outcome).

In mediation, subgroup, and Cox regression analyses, multiple adjusted models were used to ensure robustness: Model I was adjusted for age, sex, BMI, hypertension, diabetes mellitus, smoking, and previous PCI; Model II was adjusted for age, BMI, diabetes mellitus, AMI, heart rate, fibrinogen (Fib), bSS, LVEF, diuretics, and insulin; and Model III was adjusted for age, sex, BMI, hypertension, diabetes mellitus, smoking, previous PCI, AMI, heart rate, Fib, baseline SYNTAX score (bSS), LVEF, diuretics, and insulin.

All statistical analyses were performed with SPSS 24.0 (IBM, Armonk, New York, NY, USA), R Programming Language 4.0.2, Stata/MP 16.0 software, and MedCalc19.1 (MedCalc software, Ostend, Belgium). All tests were two-sided, and a *p*-value < 0.05 was considered statistically significant.

## 3. Results

### 3.1. Baseline Characteristics

**Figure 1:**
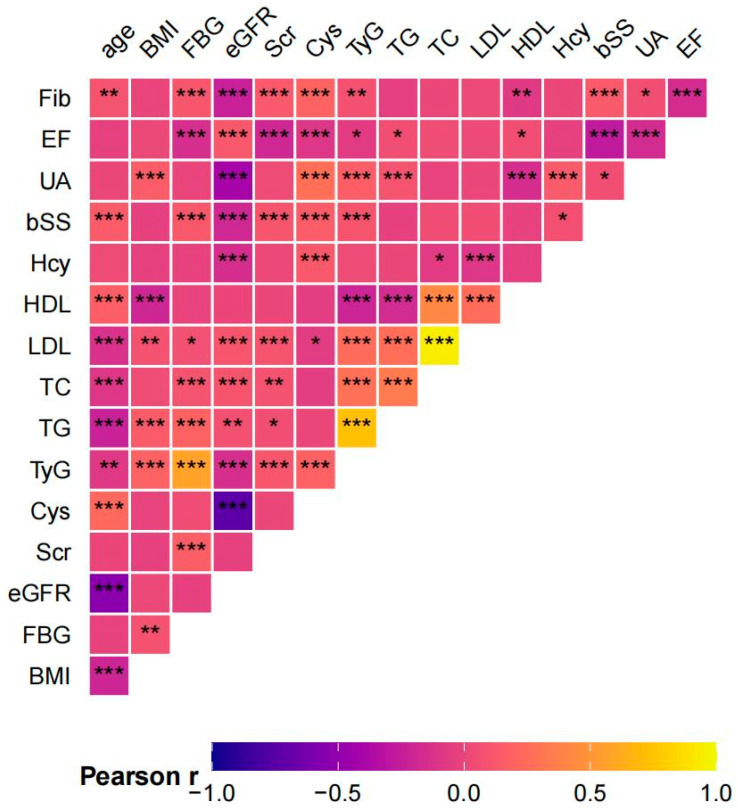
A heatmap illustrating the correlation between different variables. BMI, body mass index; UA, uric acid; FBG, fasting blood glucose; TG, triglyceride; TC, total cholesterol; HDL, high-density lipoprotein cholesterol; LDL, low-density lipoprotein cholesterol; Fib, fibrinogen; Hcy, homocysteine; EF, left-ventricular ejection fraction; Scr, serum creatinine; Cys, cystatin C; Fib, fibrinogen; eGFR, estimated glomerular filtration rate; TyG index, the triglyceride–glucose index; and bSS, baseline SYNTAX score. The intensity of color reflects the strength of the correlation. * *p* < 0.05, ** *p* < 0.01, and *** *p* < 0.001.

Ultimately, a total of 1340 eligible ACS patients were included. The average age of participants was 67.02 ± 11.21 years, with 956 (71.34%) males. Based on the occurrence of the primary endpoint, patients were divided into the MACEs group (*n* = 124) and non-MACEs group (*n* = 1216). The demographic characteristics and clinical baseline characteristics of patients in the two groups are shown in Table 1, and patients who experienced MACEs were significantly older, and had higher rates of COPD, diabetes mellitus, and myocardial infarction. They also exhibited significantly higher levels of cardiac troponin T (cTnT), B-type natriuretic peptide (BNP), uric acid, serum creatinine, cystatin C, homocysteine (Hcy), Fib, baseline SYNTAX score (bSS), and TyG index values (all *p* < 0.05). Conversely, eGFR and LVEF were significantly lower in the MACEs group (all *p* < 0.05). Furthermore, a heatmap demonstrated a significant negative relationship between the TyG index and eGFR (*p* < 0.001; Figure 1).

### 3.2. Association Between TyG Index, eGFR, and the Incidence of MACEs

Over a median follow-up of 31.02 (27.34, 35.03) months, 124 (9.25%) patients experienced MACEs. Among the patients, 88 patients experienced all-cause death, 55 patients experienced cardiac death, 47 patients experienced stroke, 40 patients experienced myocardial infarction, and 120 patients experienced unplanned revascularization. Compared with patients with a lower TyG index (<9.05), those with a higher TyG index (≥9.05) had a significantly higher incidence of MACEs, all-cause death, and cardiac death. In contrast, lower eGFR (<60 mL/min/1.73 m^2^) was significantly associated with a higher incidence of MACEs, all-cause death, and cardiac death (all *p* < 0.05, Table S1).

**Figure 2:**
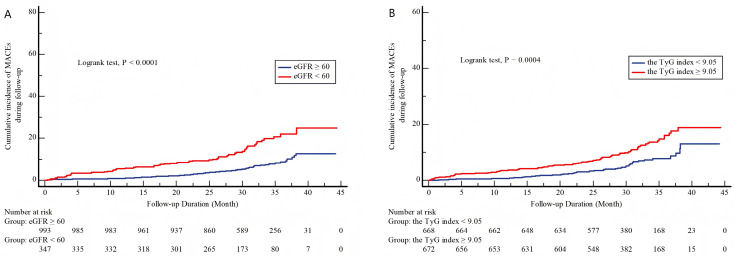
Cumulative incidence of MACEs during follow-up stratified by eGFR and the TyG index. (**A**) Cumulative incidence of MACEs during follow-up stratified by eGFR. (**B**) Cumulative incidence of MACEs during follow-up stratified by TyG index. MACEs, major adverse cardiovascular events; TyG index, the triglyceride–glucose index; and eGFR, estimated glomerular filtration rate.

**Figure 3:**
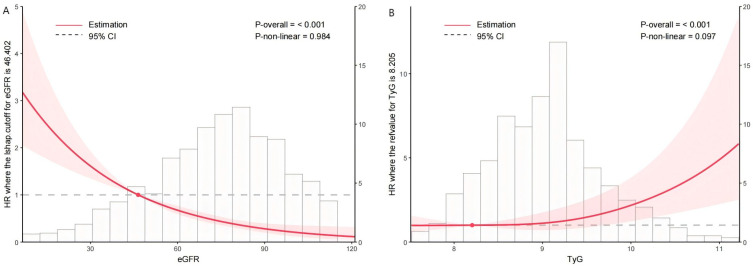
Dose–response relationship of the eGFR and TyG index with the risk of MACEs in ACS-undergoing PCI. (**A**) Dose–response relationship of the eGFR with the risk of MACEs in ACS-undergoing PCI. (**B**) Dose–response relationship of the TyG index with the risk of MACEs in ACS-undergoing PCI. The restricted cubic spline (RCS) curves are derived from a Cox regression adjustment model, which includes factors such as age, BMI, diabetes mellitus, eGFR, Fib, heart rate, bSS, diuretics, Isu, AMI, and LVEF. eGFR, estimated glomerular filtration rate; TyG index, the triglyceride–glucose index; HR, hazard ratio; and CI, confidence interval.

Kaplan–Meier analysis confirmed that lower eGFR and higher TyG index were significantly associated with a higher cumulative incidence of MACEs in ACS patients (all *p* < 0.05, Figure 2). Restricted cubic spline (RCS) analysis revealed a linear inverse relationship between eGFR and MACEs risk and a positive dose–response relationship between TyG index and MACEs risk (Figure 3).

### 3.3. Univariable and Multivariable Cox Regression Analyses

Univariate Cox regression indicated that age, AMI, diabetes mellitus, heart rate, serum creatinine, cystatin C, eGFR, fibrinogen, bSS, TyG index, LVEF, and the use of diuretics and insulin were significantly associated with the risk of MACEs (all *p* < 0.05, Table S2).

In multivariate analysis, both an elevated TyG index (unadjusted model: HR 1.620, 95% CI 1.301–2.016, *p* < 0.001; Model I: HR 1.731, 95% CI 1.282–2.337, *p* < 0.001; Model II: HR 1.728, 95% CI 1.278–2.336, *p* < 0.001; and Model III: HR 1.738, 95% CI 1.273–2.372, *p* < 0.001) and a declining eGFR (unadjusted model: HR 1.338, 95% CI 1.125–1.432, *p* < 0.001; Model I: HR 1.205, 95% CI 1.110–1.309, *p* < 0.001; Model II: HR 1.128, 95% CI 1.035–1.230, *p* = 0.006; and Model III: HR 1.127, 95% CI 1.032–1.232, *p* = 0.008) were independently associated with an increased risk of MACEs. Similar associations were observed for all-cause death (*p* < 0.05, Table 2).

### 3.4. The Predictive Value of the TyG Index and eGFR for MACEs in Various Subgroups

**Figure 4:**
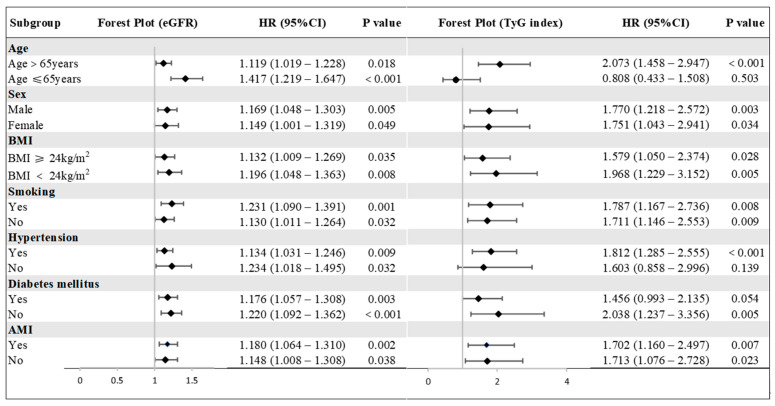
The association of the eGFR and the TyG index with the risk of MACEs in ACS-undergoing PCI stratified by different subgroups. eGFR, estimated glomerular filtration rate; TyG index, the triglyceride–glucose index; BMI, body mass index; AMI, acute myocardial infarction; and CI, confidence interval. Hazard ratio (HR) indicates an increased risk for each 10-unit decrease in eGFR. All models were adjusted for age, BMI, diabetes mellitus, AMI, heart rate, Fib, bSS, LVEF, diuretics, and insulin.

Subgroup analyses assessed the consistency of the predictive value of the TyG index and eGFR across various patient characteristics stratified by age, sex, BMI, smoking status, diabetes, hypertension, and AMI (Figure 4). After multivariable adjustment, eGFR remained a significant predictor of MACEs across all subgroups (all *p* < 0.05). The TyG index was significantly associated with MACEs risk in patients regardless of sex, BMI, smoking status, or AMI diagnosis (all *p* < 0.05). In other subgroups, the TyG index was significantly associated with MACEs risk only in patients aged over 65 years, those with hypertension, or those without diabetes (all *p* < 0.05).

### 3.5. Mediation Analysis

**Figure 5:**
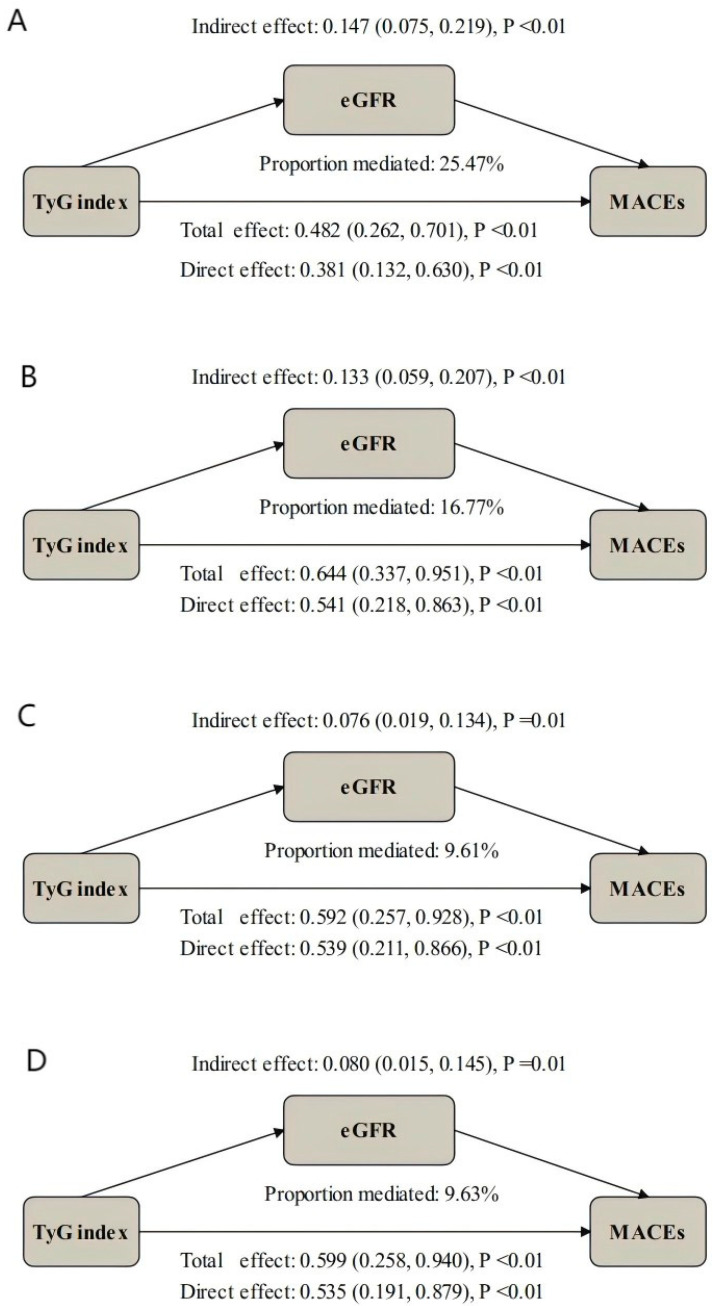
The role of eGFR in mediating the association between the TyG index and MACEs. Model I was adjusted for age, sex, BMI, hypertension, diabetes mellitus, smoking, previous PCI; Model II was adjusted for age, BMI, diabetes mellitus, AMI, heart rate, Fib, bSS, LVEF, diuretics, insulin; and Model III was adjusted for age, sex, BMI, hypertension, diabetes mellitus, smoking, previous PCI, AMI, heart rate, Fib, bSS, LVEF, diuretics, insulin. Hazard ratio (HR) indicates an increased risk for each 10-unit decrease in eGFR. CI, confidence interval; eGFR, estimated glomerular filtration rate; MACEs, major adverse cardiovascular events; MI, myocardial infarction, and TyG index, the triglyceride–glucose index.

Given the independent associations of both TyG and eGFR with MACEs and their significant correlation with each other, we further investigated whether renal function mediates this relationship. Mediation analysis demonstrated that eGFR significantly partially mediated the relationship between the TyG index and MACEs incidence across all adjusted models (Table 3; Figure 5). The mediation proportions of eGFR were 25.47% (95%CI: 13.52, 53.90%, *p* < 0.01), 16.77% (CI: 7.75, 33.64%, *p* < 0.01), 9.61% (CI: 1.17, 23.79%, *p* = 0.02), and 9.63% (CI: 1.77, 23.40%, *p* = 0.01) in the unadjusted, adjusted Model I, adjusted Model II, and adjusted Model III analyses, respectively.

## 4. Discussion

This retrospective cohort study demonstrates that both an elevated baseline TyG index and a reduced level of eGFR are significantly associated with a higher incidence of MACEs in patients with ACS after PCI—even after adjusting for potential confounders. Importantly, reduced eGFR partially mediated the relationship between the TyG index and the hazard of MACEs. These findings suggest that IR exacerbates adverse cardiovascular outcomes in ACS patients, at least in part, through the deterioration of renal function.

IR, as a core feature of systemic metabolic disorder, is directly involved in the progression of coronary atherosclerosis [3]. It drives the formation and progression of atherosclerotic plaques through impaired endothelial function [25], activated inflammatory responses [26], and dyslipidemia [27,28]. The TyG index, a simple and economical surrogate marker of IR [6], is closely related to cardiovascular and cerebrovascular diseases [29]. The China Cardiovascular Disease Medical Quality Improvement Project-Acute Coronary Syndrome (CCC-ACS) multicenter cohort study revealed that in ACS patients, the TyG index was significantly positively correlated with the risk of in-hospital adverse cardiovascular outcomes after PCI [30]. Similarly, studies based on the US National Health and Nutrition Examination Survey (NHANES) and the Iranian Isfahan cohort demonstrated a dose–response relationship between the TyG index and all-cause mortality in general coronary heart disease populations [31,32], supporting its utility as a cross-population risk marker. Our results align with these studies, showing a strong positive association between the TyG index and MACEs across multiple models and subgroups. Each one-unit increase in the TyG index was associated with a 73.8% increase in MACEs risk in the fully adjusted model. Furthermore, the predictive performance of the TyG index varied across subgroups. It was significantly associated with MACEs in patients older than 65 years but not in younger individuals, possibly due to age-related declines in metabolic reserve and amplified cardiovascular damage from IR [33,34]. Additionally, the TyG index has a significantly strong risk prediction for non-diabetic populations, but not for diabetic populations, likely due to the ubiquitous presence of IR in diabetic populations which may diminish the discriminative power of the TyG index [35].

Epidemiological evidence consistently demonstrates that renal impairment correlates with the severity of coronary artery disease, increased risks of in-hospital cardiac death, and long-term all-cause mortality in ACS patients [17,18]. Consistent with previous studies, our study revealed that eGFR was significantly negatively associated with the hazard of MACEs in ACS patients, with each ten-unit decrease in eGFR corresponding to a 12.7% increase in risk of MACEs. The relationship involves multiple pathways. First of all, renal impairment promotes cardiovascular damage through the accumulation of uremic toxins (such as urea, systemic inflammatory factors, p-cresyl sulfate and indoxyl sulfate) which induce oxidative stress, inflammation, and endothelial dysfunction, thereby accelerating atherosclerosis and plaque destabilization [36,37,38,39,40,41]. Secondly, renal insufficiency leads to lipid metabolism disorder, characterized by increased triglycerides, LDL and VLDL levels, decreased HDL-C, or carbamoylation of HDL, which induces cholesterol accumulation in macrophages and accelerates atherosclerotic lesion progression [42,43]. Furthermore, renal failure can accelerate CAD by the renin–angiotensin–aldosterone system (RAAS) [44,45], pre-calcification pathway [38], advanced glycation end-products (sRAGE) [46], and intestinal microbiome [47] and IR, promoting the progression of coronary atherosclerosis or acute cardiovascular events [38].

Additionally, we found a significant negative correlation between the TyG index and eGFR. IR contributes to peripheral microvascular dysfunction [16], and the kidney is a major target of microvascular damage in metabolic diseases. Under IR conditions, increased glucose load in renal tubules activates the tubuloglomerular feedback mechanism [20], promoting abnormal glomerular hemodynamics [48,49]. Concurrently, lipotoxicity induces podocyte apoptosis and renal tubular interstitial fibrosis through oxidative stress, endoplasmic reticulum stress, mitochondrial dysfunction, and inflammation [50], collectively leading to progressive and irreversible nephron damage and reduced eGFR.

Most importantly, mediation analysis confirmed that renal impairment significantly mediates part of the relationship between IR and MACEs risk, consistent with findings from another national cohort study [21]. The mediation analysis revealed that renal impairment accounted for 9.63% of the total TyG–MACEs association, even after adjusting for confounders. This indicates that insulin resistance contributes to cardiovascular risk not only directly through metabolic effects on the heart but also indirectly by promoting renal impairment. This finding further validates the concept of “cardio-kidney metabolic syndrome”, where metabolic disturbances, renal impairment, and cardiovascular damage form a vicious cycle which leads to multiorgan dysfunction [51], significantly amplifying the adverse outcomes risk in ACS patients. Practically and clinically, serial monitoring of the TyG index and eGFR may enhance risk stratification in ACS patients post-PCI. For high-risk populations, combined therapeutic strategies targeting IR and renal protection such as using GLP-1 agonists and sodium–glucose cotransporter-2 inhibitors [52] may have greater clinical benefits.

### Strength and Limitations

This study employs rigorous statistical methods to quantify the mediating role of renal impairment in the association between insulin resistance and long-term MACEs in ACS patients. This study’s finding provides a pathophysiological perspective on how metabolic disorders contribute to cardiovascular risks in high-risk populations, transcending the limitations of traditional correlation analyses. Furthermore, the study is based on a sizable cohort (*n* = 1340) with a relatively long and complete follow-up (median 31.02 months), and all endpoints were rigorously adjudicated against medical records, enhancing the reliability of the results.

However, several limitations should be acknowledged. First, as this study employed a retrospective design, despite adjustment for extensive clinical and laboratory confounders, it may still be subject to residual confounding due to dietary habits, genetic predisposition, or medication adherence. Second, our assessment of renal function relied on eGFR, without incorporating more sensitive and specific biomarkers of early renal damage (such as neutrophil gelatinase-associated lipocalin [53] and kidney injury molecule-1 [54]). This might have led to an underestimation of the true mediating effect of renal impairment in the pathway between TyG index and MACEs. Third, the study population was recruited from a single center in China, which may limit the generalizability of our findings to other ethnic or healthcare settings. Therefore, it is necessary to carry out prospective, multicenter studies that include novel renal biomarkers, and dynamic measurements of both TyG index and eGFR are warranted to validate and extend our conclusions. Additionally, further research should explore other potential mediators in the pathway between IR and cardiovascular outcomes to provide a more comprehensive understanding of their underlying mechanisms.

## 5. Conclusions

Our study demonstrates that renal impairment is a significant partial mediator in the association between IR and adverse cardiovascular outcomes in ACS patients. This finding underscores the importance of the metabolic–cardiorenal axis in ACS patients. A comprehensive assessment and management strategy addressing both IR and renal function may improve risk stratification and therapeutic outcomes for this high-risk population.

## Figures and Tables

**Table 1:** Baseline characteristics stratified by the incidence of MACEs.

Variable	Total Population	Non-MACEs (*n* = 1216)	MACEs (*n* = 124)	*p*-Value
Age, years	67.02 ± 11.21	66.34 ± 11.11	73.62 ± 10.05	**<0.001**
Female, *n* (%)	384 (28.66)	340 (27.96)	44 (35.48)	0.078
BMI, kg/m^2^	24.40 ± 2.97	24.45 ± 2.96	23.91 ± 2.95	0.050
Smoking, *n* (%)	715 (53.36)	658 (54.11)	57 (45.97)	0.083
Previous PCI, *n* (%)	115 (8.58)	101 (8.31)	14 (11.29)	0.258
COPD, *n* (%)	41 (3.06)	31 (2.55)	10 (8.06)	**0.001**
Hypertension, *n* (%)	911 (67.99)	818 (67.27)	93 (75.00)	0.079
Diabetes mellitus, *n* (%)	533 (39.78)	470 (38.65)	63 (50.81)	**0.008**
Stroke, *n* (%)	63 (4.70)	57 (4.69)	6 (4.84)	0.944
SBP, mmHg	132.98 ± 21.02	132.95 ± 20.81	133.22 ± 23.03	0.893
Heart rate, bpm	77.04 ± 14.29	76.73 ± 13.88	80.11 ± 17.65	**0.012**
cTnT, pg/mL	58.15 (12.33, 1211.90)	50.80 (11.60, 1211.90)	229.90 (25.75, 1919.25)	**<0.001**
BNP, pg/mL	124.75 (44.25, 328.49)	118.15 (41.85, 328.49)	328.49 (96.13, 988.13)	**<0.001**
Uric acid, µmol/L	367.05 (306.23, 434.00)	365.75 (306.10, 430.58)	384.30 (308.25, 498.10)	**0.028**
Serum creatinine, mg/dL	0.87 (0.74, 1.04)	0.87 (0.74, 1.02)	0.97 (0.78, 1.36)	**<0.001**
Cystatin C, mg/dL	1.14 (0.97, 1.41)	1.12 (0.96, 1.37)	1.37 (1.11, 2.00)	**<0.001**
eGFR, mL/min/1.73 m^2^	75.93 (59.35, 91.33)	77.87 (61.38, 92.38)	62.52 (36.52, 77.08)	**<0.001**
eGFR < 60 mL/min/1.73 m^2^	347 (25.9)	291 (23.93)	56 (45.16)	**<0.001**
FBG, mmol/L	6.95 ± 3.21	6.93 ± 3.23	7.21 ± 3.01	0.356
TG, mmol/L	1.85 ± 1.37	1.87 ± 1.41	1.63 ± 0.93	0.063
TC, mmol/L	4.47 ± 1.27	4.48 ± 1.27	4.45 ± 1.26	0.847
HDL-C, mmol/L	1.16 ± 0.30	1.16 ± 0.30	1.16 ± 0.31	0.973
LDL-C, mmol/L	2.73 ± 0.93	2.74 ± 0.93	2.73 ± 0.93	0.903
Hcy, µmol/L	13.85 (11.00, 17.40)	13.60 (10.90, 17.00)	16.65 (12.53, 20.78)	**<0.001**
Fib, g/L	3.80 ± 1.31	3.75 ± 1.27	4.24 ± 1.58	**<0.001**
LVEF	55.06 ± 8.68	55.47 ± 8.30	51.03 ± 11.03	**<0.001**
AMI, *n* (%)	667 (49.78)	591 (48.60)	76 (61.29)	**0.007**
Diagnosis, *n* (%)				**0.016**
UA	673 (50.22)	625 (51.40)	48 (38.71)	
NSTEMI	283 (21.12)	247 (20.31)	36 (29.03)	
STEMI	384 (28.66)	344 (28.29)	40 (32.26)	
Aspirin, *n* (%)	1321 (98.58)	1202 (98.85)	119 (95.97)	**0.010**
P_2_Y_12_-receptor inhibitor, *n* (%)	1319 (98.43)	1199 (98.60)	120 (96.77)	0.119
Statins, *n* (%)	1312 (97.91)	1191 (97.94)	121 (97.58)	0.788
β-blockers, *n* (%)	935 (69.78)	850 (69.90)	85 (68.55)	0.755
ACEI/ARB, *n* (%)	607 (45.30)	545 (44.82)	62 (50.00)	0.270
Diuretics, *n* (%)	230 (17.16)	185 (15.21)	45 (36.30)	**<0.001**
Insulin, *n* (%)	144 (10.75)	121 (9.95)	23 (18.55)	**0.003**
TyG index	9.05 ± 0.69	9.03 ± 0.68	9.31 ± 0.74	**<0.001**
bSS	13.00 (8.00, 20.000)	13.00 (8.00, 19.50)	19.00 (13.00, 27.85)	**<0.001**

Data are presented as mean ± SD, median (IQR), or *n* (%). Abbreviations: BMI, body mass index; PCI, percutaneous coronary intervention; COPD, chronic obstructive pulmonary disease; SBP, systolic blood pressure; cTnT, cardiac troponin T; BNP, brain natriuretic peptide; eGFR, estimating glomerular filtration rate; FBG, fasting blood glucose; TG, triglyceride; TC, total cholesterol; HDL-C, high-density lipoprotein cholesterol; LDL-C, low-density lipoprotein cholesterol; Hcy, homocysteine; Fib, fibrinogen; LVEF, left-ventricular ejection fraction; AMI, acute myocardial infarction; UA, unstable angina; STEMI, ST-segment elevation myocardial infarction; NSTEMI, non-ST-segment elevation myocardial infarction; ACEI/ARB, angiotensin-converting enzyme inhibitor/angiotensin-receptor blocker; TyG index, the triglyceride–glucose index; bSS, baseline SYNTAX score; and MACEs, major adverse cardiovascular events.

**Table 2:** Associations between the eGFR and the TyG index with the risk of MACEs and death.

		eGFR		TyG Index	
		HR (95% CI)	*p*	HR (95% CI)	*p*
MACEs	Unadjusted Model	1.338 (1.125–1.432)	**<0.001**	1.620 (1.301–2.016)	**<0.001**
	Adjusted Model I	1.205 (1.110–1.309)	**<0.001**	1.731 (1.282–2.337)	**<0.001**
	Adjusted Model II	1.128 (1.035–1.230)	**0.006**	1.728 (1.278–2.336)	**<0.001**
	Adjusted Model III	1.127 (1.032–1.232)	**0.008**	1.738 (1.273–2.372)	**<0.001**
Death	Unadjusted Model	1.366 (1.261–1.479)	**<0.001**	1.635 (1.257–2.126)	**<0.001**
	Adjusted Model I	1.220 (1.107–1.344)	**<0.001**	1.695 (1.184–2.426)	**0.004**
	Adjusted Model II	1.140 (1.028–1.264)	**0.013**	1.788 (1.241–2.574)	**0.002**
	Adjusted Model III	1.139 (1.026–1.265)	**0.015**	1.751 (1.203–2.546)	**0.003**

Model I was adjusted for age, sex, BMI, hypertension, diabetes mellitus, smoking, previous PCI; Model II was adjusted for age, BMI, diabetes mellitus, AMI, heart rate, Fib, bSS, LVEF, diuretics, insulin; and Model III was adjusted for age, sex, BMI, hypertension, diabetes mellitus, smoking, previous PCI, AMI, heart rate, Fib, bSS, LVEF, diuretics, insulin. Hazard ratio (HR) indicates an increased risk for each 10-unit decrease in eGFR. CI, confidence interval; eGFR, estimated glomerular filtration rate; MACEs, major adverse cardiovascular events; TyG index, the triglyceride–glucose index. Other abbreviations can be seen in Table 1.

**Table 3:** The role of eGFR in mediating the association between the TyG index and MACEs.

Exposures	Association							
	Total Effect		Indirect Effect		Direct Effect			
	HR (95% CI)	*p*	HR (95% CI)	*p*	HR (95% CI)	*p*	PM (95% CI)	*p*
Unadjusted Model	1.608 (1.267, 1.950)	**<0.01**	1.162 (1.079, 1.245)	**<0.01**	1.456 (1.094, 1.819)	<0.01	25.47 (13.52, 53.90)	**<0.01**
Model I	1.895 (1.309, 2.481)	**<0.01**	1.146 (1.058, 1.234)	**<0.01**	1.714 (1.159, 2.269)	<0.01	16.77 (7.75, 33.64)	**<0.01**
Model II	1.803 (1.207, 2.400)	**<0.01**	1.081 (1.013, 1.149)	**<0.01**	1.703 (1.118, 2.288)	<0.01	9.61 (1.17, 23.79)	**0.02**
Model III	1.823 (1.201, 2.444)	**<0.01**	1.082 (1.012, 1.153)	**<0.01**	1.721 (1.104, 2.337)	<0.01	9.63 (1.77, 23.40)	**0.01**

Model I was adjusted for age, sex, BMI, hypertension, diabetes mellitus, smoking, previous PCI; Model II was adjusted for age, BMI, diabetes mellitus, AMI, heart rate, Fib, bSS, LVEF, diuretics, insulin; and Model III was adjusted for age, sex, BMI, hypertension, diabetes mellitus, smoking, previous PCI, AMI, heart rate, Fib, bSS, LVEF, diuretics, insulin. HR, hazard ratio; CI, confidence interval; and PM, proportion mediated. Other abbreviations can be seen in Table 1.

## Data Availability

The datasets used and/or analyzed during the study are available from the corresponding author upon reasonable request.

## Supplementary Materials

The following supporting information can be downloaded at https://www.mdpi.com/article/10.3390/jcdd12110427/s1. Table S1. Comparison of Long-Term Adverse Prognosis Stratified by eGFR and TyG index Categories. Table S2. Univariate Cox regression analysis for MACEs.
