# Supplementary material for: The Synergistic Risk of Insulin Resistance and Renal Dysfunction in Acute Coronary Syndrome Patients After Percutaneous Coronary Intervention
Source: J Cardiovasc Dev Dis. 2025 Oct 28;12(11):427. doi: 10.3390/jcdd12110427 (PMC12653602; doi:10.3390/jcdd12110427)
Supplement: Supplementary file 1 [file jcdd-12-00427-s001.zip › jcdd-3893159-supplementary.pdf]

Supplementary data

Scheme 1. (Table S1) Comparison of Long-Term Adverse Prognosis Stratified by eGFR and TyG index Categories.

Variable	eGFR		P	TyG index		P
	**eGFR ≥60 (n=993)**	eGFR <60 (n=347)		TyG index <9.05 (n=668)	TyG index ≧9.05 (n=672)	
**MACEs, n (%)**	**68 (6.85)**	56(16.14)	**< 0.001**	43(6.44)	81(12.05)	**< 0.001**
All-cause death, n (%)	44 (4.43)	44(12.68)	**< 0.001**	28(4.19)	60(8.93)	**< 0.001**
Cardiac death, n (%)	26 (2.62)	29(8.36)	**< 0.001**	15(2.25)	40(5.95)	**0.001**
MI, n (%)	28 (2.82)	12(3.46)	0.547	17(2.54)	23(3.42)	0.345
Unplanned revascularization, n (%)	86 (8.66)	34(9.80)	0.523	64(9.58)	56(8.33)	0.424
Stroke, n (%)	34 (3.42)	13(3.75)	0.779	24(3.59)	23(3.42)	0.866

MACEs refer to major adverse cardiovascular events, defined as a composite outcome of all-cause death, nonfatal myocardial infarction (MI). TyG index, the triglyceride–glucose index; eGFR, estimated glomerular filtration rate;.

Scheme 2. (Table S2) Univariate Cox regression analysis for MACEs.

**Variables**	**Univariate analysis**		
	**HR**	**95%CI**	**P**
Age	1.070	1.050 - 1.090	**<0.001**
Female	1.389	0.961 - 2.007	0.080
BMI	0.932	0.877 - 0.991	**0.024**
AMI	1.660	1.157 - 2.383	**0.006**
Smoking	0.745	0.523 - 1.061	0.102
Previous PCI	1.325	0.760 - 2.312	0.321
Hypertension	1.418	0.945 - 2.130	0.092
Diabetes Mellitus	1.610	1.132 - 2.290	**0.008**
SBP	1.000	0.991 - 1.008	0.958
Heart rate	1.016	1.004 - 1.028	0.007
Serum creatinine	1.180	1.093 - 1.275	**<0.001**
Cystatin C	1.416	1.285 - 1.560	**<0.001**
eGFR	0.971	0.965 - 0.978	**<0.001**
FBG	1.024	0.974 - 1.077	0.349
TC	0.980	0.852 - 1.127	0.774
TG	0.837	0.699 - 1.002	0.052
HDL-C	1.028	0.576 - 1.834	0.927
LDL-C	0.968	0.799 - 1.174	0.744
Hcy	1.002	0.996 - 1.008	0.511
Fib	1.221	1.110 - 1.344	**<0.001**
bSS	1.056	1.038 - 1.073	**<0.001**
TyG index	1.620	1.301 - 2.016	**<0.001**
LVEF	0.955	0.939 - 0.971	**<0.001**
β-blockers	0.930	0.637 - 1.360	0.709
Diuretics	2.977	2.064 - 4.293	**<0.001**
ACEI/ARB	1.182	0.831 - 1.681	0.353
Insulin	1.950	1.240 - 3.067	**0.004**

BMI, body mass index; AMI, acute myocardial infarction; PCI, percutaneous coronary intervention; SBP, systolic blood pressure; FBG, fasting blood glucose; TG, triglyceride; TC, total cholesterol; HDL-C, high density lipoprotein cholesterol; LDL-C, low density lipoprotein cholesterol; eGFR, estimated glomerular filtration rate; Fib, fibrinogen; Hcy, Homocysteine; LVEF, left ventricular ejection fraction; ACEI/ARB, angiotensin converting enzyme inhibitor/angiotensin receptor blocker;

TyG index, the triglyceride–glucose index; bSS, baseline SYNTAX score; HR, hazard ratio; CI, confidence interval.
